# Decision Points: The Factors Influencing the Decision to Feed in the Medicinal Leech

**DOI:** 10.3389/fnins.2012.00101

**Published:** 2012-07-06

**Authors:** Quentin Gaudry, William B. Kristan

**Affiliations:** ^1^Department of Neurobiology, Harvard Medical SchoolBoston, MA, USA; ^2^Neurobiology Section, Division of Biological Sciences, University of California San DiegoLa Jolla, CA, USA

**Keywords:** behavioral choice, decision-making, distributed, feeding, leech, modular, sensory gating, serotonin

## Abstract

The decision to feed is a complex task that requires making several small independent choices. Am I hungry? Where do I look for food? Is there something better I’d rather be doing? When should I stop? With all of these questions, it is no wonder that decisions about feeding depend on several sensory modalities and that the influences of these sensory systems would be evident throughout the nervous system. The leech is uniquely well suited for studying these complicated questions due to its relatively simple nervous system, its exceptionally well-characterized behaviors and neural circuits, and the ease with which one can employ semi-intact preparations to study the link between physiology and decision-making. We will begin this review by discussing the cellular substrates that govern the decision to initiate and to terminate a bout of feeding. We will then discuss how feeding temporarily blocks competing behaviors from being expressed while the animal continues to feed. Then we will review what is currently known about how feeding affects long-term behavioral choices of the leech. Finally, we conclude with a short discussion of the advantages of the leech’s decision-making circuit’s design and how this design might be applicable to all decision circuits.

## Introduction

To survive and obtain the necessary energy to fuel everyday life, animals must feed. This universal drive makes feeding an ideal system for studying decision-making processes within the nervous system. The decision to feed involves key decision points such as how to locate a food source, the decision to initiate a bout of feeding, whether or not to continue feeding given competing external stimuli, and when to terminate feeding. In this review, we will consider behavioral choice as a form of decision-making. In general, behavioral choice means that an animal has more than one stimulus or behavioral state to which it may respond. Second, and most importantly, the animal responds to just one of them (Sherrington, 1906; Kovac and Davis, 1977; Davis, 1979; Everett et al., 1982; Misell et al., 1998). In the experiments described within, leeches were presented with either multiple stimuli (such as food and tactile stimulation) or the same stimulus while in different behavioral states (such as satiated or hungry). In such cases, we will describe the ensuing motor pattern, or lack thereof, as the leech’s behavioral “choice.” The neuronal mechanisms leading up to this choice will be described as the “decision-making process.”

The nervous system of the leech is an ideal system for studying the neuronal substrates of decision-making, particularly for feeding. First, the leech nervous system is relatively small with only about 400 unique neurons reiterated in its 21 segmental ganglia (Macagno, 1980). Second, most of these neurons are identifiable from preparation to preparation, which makes studying their role in decision-making far simpler than sampling from populations of neurons. Third, there already exists a wealth of knowledge about the feeding behavior of leeches and the circuits within their nervous system (Kristan et al., 2005). Fourth, there are many species of leeches that have evolved different feeding strategies, which makes this system an attractive model for studying how the neuronal circuits governing behaviors and decision-making processes evolve within a phylogenic clade (Lent, 1973; Keyser and Lent, 1977; Baltzley et al., 2010).

In this review, we will focus mainly on the European medicinal leech, *Hirudo verbana*, with occasional comparisons to other leech species. In general, when we refer to “the leech,” we mean *H. verbana* with our apologies to the hundreds of other leech species. We will first describe some of the factors underlying their sensation of hunger and the sensory cues that influence the decision to initiate and terminate a feeding bout. Next, we will describe the more complex interactions within the leech nervous system that prevent competing behaviors from being expressed during a feeding bout and how feeding affects their long-term behavioral choice. Then we will conclude with a brief discussion of the advantages of the design of this circuit in the leech and what this research has taught us about decision-making as a general phenomenon.

## Neuronal Mechanisms and Decision to Initiate and Terminate Feeding

The first of many decision points in feeding is the decision to initiate a feeding bout. To begin feeding requires two key elements: (1) the animal must be sufficiently motivated (i.e., it must be hungry), and (2) the proper appetitive stimuli must be present. Medicinal leeches may go a year or more between bouts of feeding (Lent and Dickinson, 1988) and serotonin levels are strongly correlated with the behavioral state of the leech (Lent et al., 1991). Well-fed or satiated leeches are typically found in deeper water and do not respond to appetitive cues such as warm objects (Dickinson and Lent, 1984). Leeches in this state have up to 28% less serotonin in their nervous system compared to hungry leeches. Removing the ingested blood from sated animals returns their serotonin levels back to levels seen in hungry leeches, and feeding behaviors resume (Lent et al., 1991). Distention not only prevents serotonin levels from returning to the levels of hungry leeches, but artificial distention also blocks 5-HT neurons from responding to appetitive stimuli as they normally do in hungry animals (Lent and Dickinson, 1987, 1988). Furthermore, injection of the toxin 5-7 D-HT depletes serotonin from leech neurons and makes hungry leeches act as though they are satiated. Soaking these toxin-treated leeches in a bath containing serotonin restores appetitive behaviors (Lent and Dickinson, 1984). These studies clearly illustrate the strong influence of serotonin on a leech’s decision to initiate feeding.

A hungry leech will feed if appropriate stimuli are present. Hungry *H. verbana* use both visual (Dickinson and Lent, 1984) and mechanical (Young et al., 1981) cues from water waves to determine whether prey is present and which direction to move. Chemical cues also promote swimming during foraging behavior (Brodfuehrer et al., 2006). Once contact is made with a potential host, both thermal and chemical cues govern the decision to feed. Leeches bite with a higher frequency to test stimuli at 38°C when either tested on a hot plate covered with parafilm^®^ wax or when exposed to a warmed feeding apparatus (Lent and Dickinson, 1984). Alternative choice assays that expose leeches to two temperatures of mammalian blood show the same temperature preference (Q. Gaudry and W. B. Kristan, unpublished observations). Along with temperature, leeches also sample the chemical composition of a potential prey using chemosensory receptors located on their dorsal lip (Elliott, 1987). Studies evaluating the chemical cues required to carry out feeding behavior to completion have revealed that only NaCl and the amino acid arginine or NaCl plus simple sugars are required (Galun and Kindler, 1966; Elliott, 1986). An interesting correlate of the decision to feed can be found as early as these primary chemosensory neurons. When appetitive stimuli are presented to the dorsal lip of the animal, an increase in neuronal firing is observed in the cephalic nerves that connect the dorsal lip to the cephalic ganglion. These action potentials likely belong to the chemosensory neurons themselves (Groome et al., 1995; Perruccio and Kleinhaus, 1996). Combining aversive chemical agents to these appetitive stimuli suppresses the chemosensory activity in the cephalic nerves (Li et al., 2001). These data suggest that the integration of appetitive and non-appetitive cues may occur as early as the periphery and that the central nervous system may not have to weigh these conflicting chemical cues against each other. While this result is surprising, a similar observation occurs in the CO_2_ olfactory receptor neurons (ORNs) of the fruit fly *Drosophila melanogaster*. CO_2_ is highly aversive to this fly (Jones et al., 2007). However, CO_2_ is also found in ripened fruit, a favorite food of fruit flies. Extracellular recordings from the CO_2_ sensitive ORNs reveals that these receptors are inhibited when CO_2_ is combined with odors that co-occur in ripening fruit (Turner and Ray, 2009). Thus CO_2_ behavioral aversion is inhibited in the context of feeding. The decision to escape or to feed in this fly appears to be governed, at least in part, directly at the level of the sensory receptor.

The leech must not only decide when to start feeding, but also when to stop. There are at least two distinct sensory stimuli that are known to effectively terminate ingestion in leeches. The first is a change in the chemical quality of the food being ingested. In addition to the external chemosensory receptors mentioned above, the leech also possesses receptors that are located in its gut. These serve to continuously sample the quality of food being ingested (Kornreich and Kleinhaus, 1999). Switching feeding solutions to an aversive agent (such as quinine, denatonium, or water) quickly terminates a feeding bout. The same result is observed when these chemicals are injected into the gut of a feeding leech, thus reducing the likelihood that these chemicals came into contact with the external chemosensory neurons of the dorsal lip. The second well-described stimulus that terminates feeding is distention of the leech due to the large volume of the blood meal (Lent and Dickinson, 1987). The termination of feeding by distention is likely mediated by stretch receptors located either in the gut or the body wall of the animal. Removing the blood meal of a leech through cannulation will increase the duration of ingestion near indefinitely, thus ruling out fatigue as a meaningful cue to terminate feeding (Lent and Dickinson, 1987). Additionally, distending a leech with a saline solution is sufficient to disrupt ingestion and suggests that chemical cues may also not be necessary for signaling the leech to stop feeding. The role of distention in terminating a feeding bout is well documented among other animal groups as well, particularly in the insects (Chapman and de Boer, 1995) and mollusks (Kuslansky et al., 1987).

## Short-Term Inhibition of Competing Behaviors

The decision to feed is generally not made in the context of appetitive stimuli alone, but also in the presence of competing non-appetitive stimuli. For sanguivorous leeches, this decision is highly predictable: when a hungry sanguivorous leech detects food-related chemical cues, feeding takes precedence over all other behaviors (Gaudry et al., 2010). These animals will even ignore noxious stimuli until they obtain a full meal. Tactile stimulation of the leech normally results in a number of behaviors (Kristan et al., 1982) that are mutually exclusive with the ingestion of a blood meal. These behaviors include the locomotory behaviors, such as swimming and crawling away from the source of stimulation, or shortening, which is a rapid withdrawal of the head. Just prior to and during feeding, these behaviors are robustly inhibited (Misell et al., 1998). Furthermore, largely dissected animals and reduced preparations will display robust feeding behavior despite the trauma imposed on them during surgery (Lent and Dickinson, 1987; Wilson et al., 1996; Wilson and Kleinhaus, 2000; Gaudry and Kristan, 2009). Leeches also appear to be insensitive to aversive chemical stimuli while feeding. While a non-feeding leech will retract and pull away when exposed to denatonium or quinine (Li et al., 2001), a feeding leech will ignore these chemicals when they are presented to their external chemoreceptors located on the dorsal lip (Kornreich and Kleinhaus, 1999).

**Figure 1:**
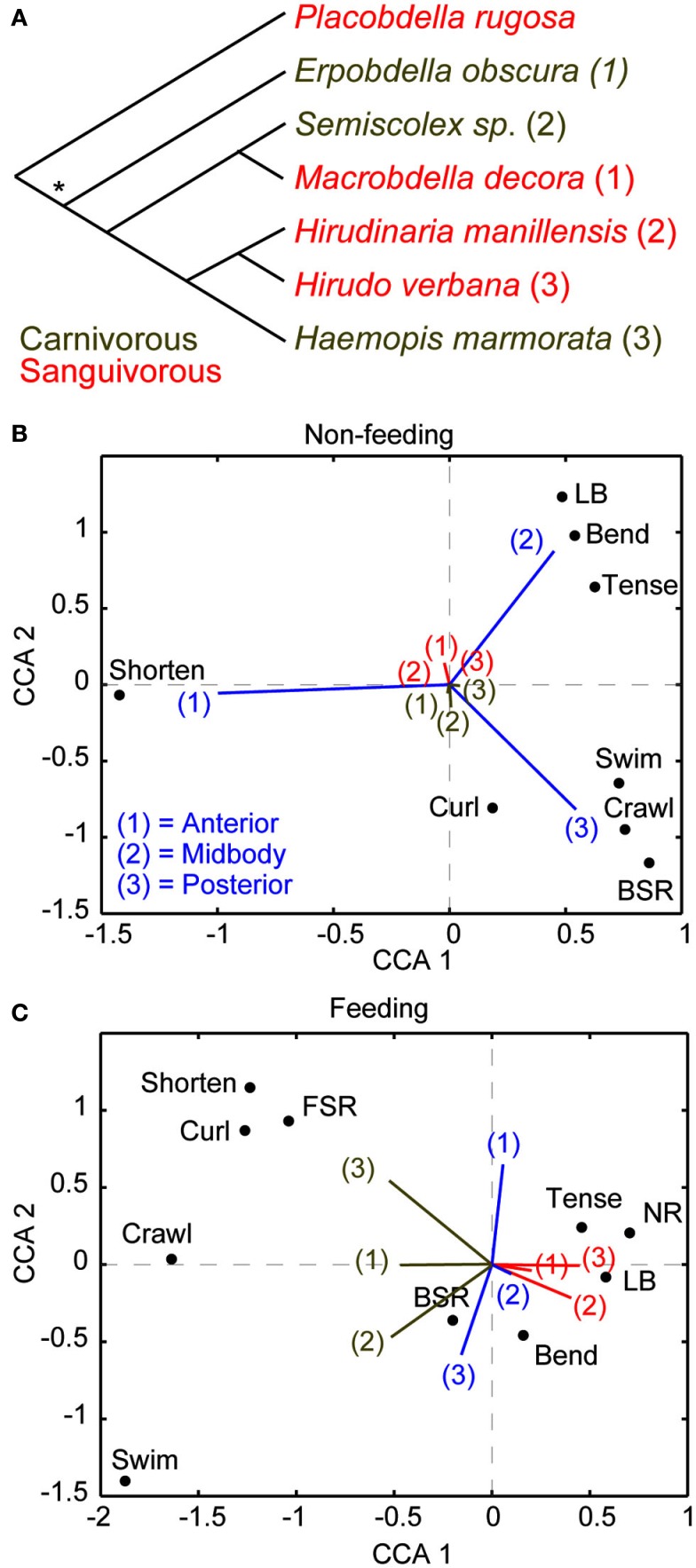
**Diet and not phylogeny determine leech behavioral choice**. **(A)** The phylogenic relationship of leeches used for this study (Gaudry et al., 2010) is based on a comparison of morphological and molecular features (Borda and Siddall, 2004). An asterisk implies an ancestral state of unknown feeding preference. The most parsimonious explanation of these relationships is that the sanguivorous feeding strategy evolved three different times among these species from a carnivorous ancestor. The numbers following each species is used to reference that species in **(B,C)**. **(B)** Canonical Correspondence Analysis (CCA) biplot showing the relationship between species, stimulus location, and behavioral output in the non-feeding state. Species and stimulus location serve as predictors and the magnitude of their vectors denotes the influence they have on the raw variables (behavioral outputs). The predictor vectors point toward the behaviors they are most strongly correlated with. The clustering of all species at the middle means that all species responded to all stimuli in similar ways. Thus species has little predictive power over the resulting behavior while stimulus location is a good indicator of which behavior will be elicited in response to stimulation in the non-feeding state. Coloring and numberings as in **(A)** where brown refers to carnivorous species and red refers sanguivorous species. **(C)** CCA results for the same group of leeches as in **(B)** but during the feeding state. The carnivorous leech vectors shown in brown point toward active behaviors [shortening, swimming, crawling, and back sucker release (BSR)] whereas the sanguivorous leech vectors shown in red point in the direction of local responses [Bend, Tense, local bend (LB)] or no response (NR). The results indicate that the diet of the leeches (regardless of their phylogenetic relationship) is the best predictor of stimulus response during feeding. (More details about CCA are found in Gaudry et al., 2010).

Before discussing the cellular substrates that underlie the suppression of noxious stimuli while feeding in leeches, we feel compelled to first ask, “why does the medicinal leech behave in this manner?” From our own experiences, we would avoid harm at the cost of a single meal. For the leech, however, a meal may come along only rarely and the leech makes the most of each opportunity by consuming a huge meal that can sustain it for up to a year. In fact, such dominance of feeding over escape responses may be a common feature among obligate sanguivores that feed at a low frequency. For instance, hard ticks (*Ixodidae*) place a similar premium on feeding and mated female ticks can gain an astounding 11,000% their body weight in a single meal and wait a full year between meals (Sonnenshine, 1991). And like medicinal leeches, they are capable of ignoring tremendous physical torment including burning and exposure to alcohol to keep feeding (Needham, 1985). Sanguivorous leeches reliably consume large meals that increase their weight by more than 800%, and mechanical stimulation of these leeches while feeding does not affect the duration of a meal or the weight gained (Gaudry et al., 2010). Among different species of leeches, diet is strongly correlated with the priority of feeding (Figure 1). Because sanguivory and carnivory have probably evolved independently several times within the leech lineage (Figure 1A; Borda and Siddall, 2004), the correlation between sanguivory and behavioral choice is more likely to be due to the diet of a species rather than its place in phylogeny. Canonical correspondence analysis (CCA) was recently used to study the relationship between leech species, feeding, and behavioral choice in detail (Gaudry et al., 2010). CCA is an analytical technique that was initially developed in the field of ecology but has also proven useful for studying the relationship between stimuli, manipulations, and behavior (Cornford et al., 2006). Similar to the more popular principle component analysis (PCA), CCA allows one to see trends in large multi-dimensional data sets by reducing the dimensionality of these data and producing biplots that highlight the relationship between important variables (Braak Ter, 1986). Unlike PCA, which is most appropriately applied to continuous and monotonic data, CCA is best applied to discrete data that can vary either linearly or unimodally. Six species of leeches (three carnivorous and three sanguivorous) were tested for their responses to tactile stimuli prior feeding. All six species responded similarly: they mostly shortened to touches at the anterior end, bended their bodies in a variety of ways when touched in the middle, and locomoted (swam or crawled) when touched at the posterior end (Figure 1B). The responses to the same stimuli were strongly curtailed by feeding in all three sanguivorous species tested, but were not changed in the carnivorous species (Figure 1C). It will be of great interest in the future to determine how the nervous systems of the carnivorous and sanguivorous leeches differ to gain a better understanding of how decision-making circuits may have evolved.

**Figure 2:**
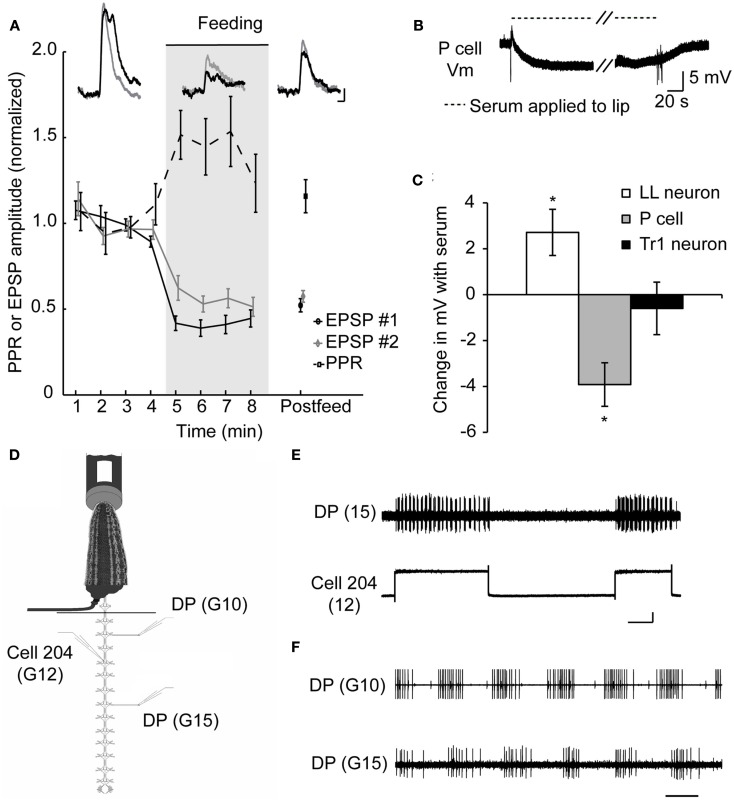
**Evidence for presynaptic inhibition of pressure mechanosensory P cells**. **(A)** Effects of feeding on EPSP amplitudes and PPR at the P cell-to-AP neuron synapse. Inserts show overlapping pairs of traces from one such experiment, sampled from pre-feeding, feeding, and post-feeding times. In each pair, the black trace is the first EPSP and the gray trace is the second EPSP in response to a P cell spike triggered 500 ms after the first one. Scale bars represent 2 mV and 50 ms (from Gaudry and Kristan, 2009). **(B)** Recording from a P cell in the leech head brain while blood serum was applied to the isolated lip of a semi-intact preparation similar to Groome et al. (1995). Diagonal dashes denote a break in the sample trace corresponding to ∼3 min. Fast vertical deflections in voltage trace are artifacts of switching the solution on at the lip of the preparation. **(C)** P cells of the cephalic or head brain are hyperpolarized when blood serum is applied. As controls we show that neurons capable of triggering swimming (Tr1) remain unaffected while the serotonergic motor effector LL cell depolarizes as described previously by others. *p < 0.05, *N* = 5 leeches. **(D)** A schematic diagram of semi-intact feeding preparation showing the sites of intracellular stimulation and extracellular recordings. Leeches were fed on warmed bovine serum. Dorsal posterior nerves (DP) contain the axon of a dorsal excitor motor neuron (DE-3) and spiking indicated the dorsal contraction phase of each swim cycle. **(E)** Depolarization of cell 204 with a 2-nA current elicited a swim pattern in the DP recorded in ganglion 15. Traces were recorded while the anterior end of the leech was feeding from the serum tube. The vertical scale bar represents 50 mV and the horizontal scale bar represents 5 s. Cell 204 spikes are small (∼5 mV) and are obscured by the relatively large depolarization caused by an inability to completely offset the electrodes resistance while passing large currents. **(F)** DP nerve recordings made anterior and posterior to the impaled 204 cell in **(E)**. Scale bar represents 500 ms. (Data from Gaudry and Kristan, 2009).

So how do sanguivorous leeches block out competing stimuli while feeding? To determine how the nervous system of a sanguivorous leech prevents mechanosensory stimuli from eliciting feeding-incompatible behaviors, we used a previously described semi-intact preparation (Wilson et al., 1996) that allows intracellular recordings to be made from the central nervous system while the rest of the animal is free to behave and most importantly, feed. These experiments revealed that the excitatory postsynaptic potentials (EPSPs) at the synapses between the pressure mechanosensory neurons (P cells) that encode touch stimuli and several of their targets is reduced (Figure 2A), some by more than 50% (Figure 2C; Gaudry and Kristan, 2009). Paired pulse ratios (PPRs) are a useful tool to assess whether a change in synaptic strength has a pre-synaptic component (Schulz et al., 1995). If the synaptic depression observed during feeding is the result of a postsynaptic mechanism, such as glutamate receptor modulation, we would expect each pulse in a paired pulse protocol to diminish by the same amount. Thus the ratio of the first to second pulse would stay the same before and during feeding (regardless of the absolute value of that ratio). If the depression during feeding occurs because less neurotransmitter is released pre-synaptically, more neurotransmitter should be available for release on the second pulse compared to the pre-feeding condition. This will cause the PPR to increase. Because a decrease in EPSP amplitude is observed along with an increase in the PPR at P cell synapses, the locus of plasticity is thought to be the pre-synaptic terminals of the P cell. Although, no change is observed in the intrinsic properties of the P cells in the midbody ganglia, a hyperpolarization of ∼4 mV is observed in the P cells of the leech head brain when a synthetic feeding solution is applied to the lip of a semi-intact preparation (Figures 2B,C). The hyperpolarization observed in the P cells of the cephalic ganglion was absent in midbody ganglia, probably because the cephalic P cells have a much more compressed dendritic arbor and may thus be electrotonically more compact (Yau, 1976).

Additional experiments suggest that the pre-synaptic inhibition of the tactile sensory neurons is the *only* site targeted by the ingestion phase of feeding to suppress competing behaviors. We found that stimulating downstream command-like neurons during feeding can still elicit swimming – which would normally be a behavior incompatible with feeding. Cell 204 is a potent initiator of swimming (Weeks and Kristan, 1978) and is situated only two synapses downstream from the P cells in the leech swim circuit (Brodfuehrer and Friesen, 1986). Using a semi-intact preparation capable of feeding (Figure 2D), we injected current into this neuron which elicited bouts of swimming in the posterior end of the leech (Figure 2E) while the anterior portion of the animal continued to feed (Gaudry and Kristan, 2009). These bouts of swimming were characteristic of normal leech swimming including a distinctive anterior to posterior progression (Figure 2F). This indicates that the neuronal circuit from the command-like neurons through the central pattern generator circuit to the motor neuronal firing is not affected by the inhibition generated by feeding. This pre-synaptic inhibition of sensory input functions as a form of sensory gating that diminishes the ability of mechanosensory stimuli from eliciting incompatible behaviors such as shortening, swimming, or crawling during feeding. There are two distinct advantages to this mechanism. First, it can abolish all mechanically elicited behaviors through a single target (the P cells), and second, it leaves interneurons unmodified in case they are needed to play a role in some aspect of feeding, because many leech neurons are multifunctional (Briggman and Kristan, 2006, 2008). Interestingly, the local bend reflex which would not seem to compromise the feeding movements, is nevertheless greatly diminished during feeding (Misell et al., 1998; Gaudry et al., 2010) as a consequence of this general mechanism. This decrease in local bending may be a collateral, neutral loss of a function or it may be an indication that the local bend interneurons are used as part of some component of feeding; the resolution of these possibilities awaits further study.

The inhibition of the P cells is thought to be mediated by the release of serotonin onto the P cell axon terminals (Gaudry and Kristan, 2009). Exogenous serotonin mimics the decrease observed in EPSP amplitudes and the increase in the PPR measured in the postsynaptic targets of the P cells. The reduction in excitatory drive is also observed at the level of motor output from the isolated leech ganglion. Stimulating P cells in the isolated ganglion elicits a burst of motor activity that corresponds to a local contraction in the intact animal (Lockery and Kristan, 1990a,b) and serotonin decreases this activity. Additionally, the serotonin antagonist mianserin reversed these effects both in the reduced isolated ganglion preparation as well as in the semi-intact feeding leech (Gaudry and Kristan, 2009).

Although all serotonin containing neurons within the leech ganglion have been putatively identified (Lent and Frazer, 1977; Lent and Dickinson, 1987), the source of serotonin that mediates this pre-synaptic inhibition remains a mystery. Serotonin has been shown to work in the leech nervous system in either a hormonal manner or as a common neurotransmitter (Kristan and Nusbaum, 1982), and it is not clear which mode of action causes the inhibition of the P cell terminals. Stimulation of the neurohormonal Retzius cells, the largest serotonin neurons in the leech CNS, does not mimic the depression of P cell synapses (Q. Gaudry, personal observation), but none of the remaining serotonin cells have been tested. The source and nature of this serotonin action may be complex, because serotonin has a wide variety of effects – even contradictory ones – on leech circuits and behavior. For example, serotonin has been shown to both promote and inhibit swimming behavior. This diversity in serotonin action can in some cases be explained by whether serotonin is applied to the brain or within specific regions within the segmental ganglia (Crisp and Mesce, 2003; Calviño and Szczupak, 2008). Additionally, we do not know which sensory neurons activate these serotonergic neurons, although it is likely that the lip chemoreceptors (Elliott, 1986, 1987) are a major source because the suppression of other behaviors is observed during the exploration of a potential food item even before the leech begins to feed (Gaudry and Kristan, 2009) and when full strength artificial blood is presented at ambient temperature to the lip of head-intact isolated nerve cord preparations (Brodfuehrer et al., 2006). Furthermore, chemosensory stimulation is known to activate some of the serotonergic neurons (Groome et al., 1995; Zhang et al., 2000). The neurons providing this modulation and their inputs need to be identified and studied directly.

## Feeding Induces Long-Term Changes in Behavior Choice

**Figure 3:**
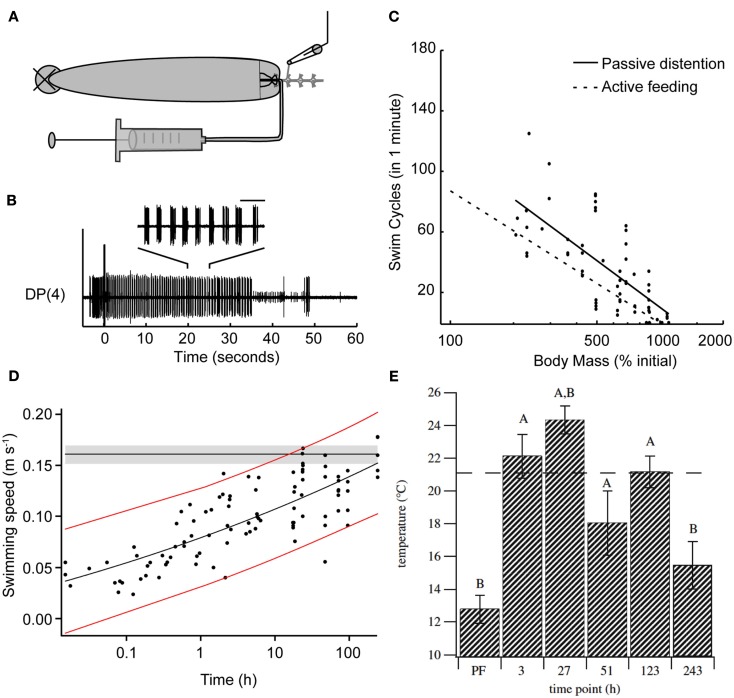
**Feeding has long-term effects on other leech behaviors**. **(A)** Schematic diagram of the semi-intact preparation used to test the impact of distention on swimming. The nerve cord was severed between ganglia 2 and 3, ganglia 3 through 5 were dissected free of the body, and extracellular recordings were made from the DP nerves of ganglion 3 or 4. Saline solution was injected via a syringe into the gut to vary the amount of distention in the intact part of the animal. **(B)** Sample trace of a DP nerve recording showing the motor neuron bursts that define swimming. The horizontal bar above inset trace corresponds to 1 s. Between each pair of bursts, the intact portion of the leech swam one complete cycle. The large stimulus artifact at time zero shows when we stimulated the body wall electrically. Motor activity that precedes the stimulus is from contact made from the stimulating electrode onto the body wall before the electrical stimulus was delivered. The inset shows an expanded view of the swim bursts between 20 and 25 s within the swim episode. **(C)** The effect of induced distention on the number of swim cycles observed within 1 min of stimulation. The *x*-axis is a logarithmic scale because this relationship appeared to be exponential. The black line is the linear regression for these data points. The dashed gray line shows best fit derived from intact active feeding preparations. [**(A–C)** from Gaudry and Kristan, 2010.] **(D)** Swimming speed measured following a bout of feeding. Leeches were fed and then stimulated to swim. The speed of each swim episode was calculated and leeches were tested for up to 10 days following feeding. Red lines represent the 95% confidence interval for post-feeding data. The horizontal black line and gray shaded area show the mean pre-feeding values and 95% confidence interval of the mean. (Modified from Claflin et al., 2009.) **(E)** Preferred temperature of leeches before and up to 10 days following feeding. Leeches were acclimated to 21°C, fed, and then tested on subsequent days. The dashed line indicates the acclimation temperature (*T*_a_). **(A)** is significantly different from the pre-feeding (PF) preferred temperature (ANCOVA, planned contrasts, Dunnett’s procedure, *p* < 0.05; *n* = 7 for PF, 3, 27 and 51 h, *n* = 6 for 123 and 243 h); **(B)** is significantly different from *T*_a_ (one-sample, two-tailed, *t*-test, *p* < 0.05 after applying Dunnett’s correction). Error bars indicate 1 SEM. (Data from Petersen et al., 2011.)

The effects of feeding on the leech’s behavioral choice extends far beyond the ingestion period. One clear result of feeding is the massive weight gain and distention that the animal experiences. Feeding strongly biases leeches away from swimming and toward crawling for at least 1 h following a meal (Misell et al., 1998), and unpublished data show that this period of suppression lasts for several days (S. Copado, Q. Gaudry, W. Kristan, Unpublished data). This bias toward crawling could be caused by one or more candidate cues: thermal, chemical, and distention. A series of experiments using semi-intact preparations point to stretch receptors likely located in the body wall of the animal as the likely decision point for biasing the animal away from swimming (Gaudry and Kristan, 2010). After severing the connections between the anterior brain and the rest of the nervous system, leeches will feed and their gut fills with the ingested fluid without any descending neuronal information. Stimulating the posterior end of such a leech reliably induces swimming behavior. As the feeding episode continues and the amount of body distention increases, swimming decreases. Removing the blood meal from the crop of the distended leech restores pre-feeding levels of swimming. Artificially distending semi-intact animals with a saline solution rapidly (in a few seconds) and reversibly inhibits swimming (Figures 3A,B). This inhibition scales logarithmically with distention (Figure 3C). Thus it is likely that distention, along with the inhibition of P cell synaptic release described above, help to inhibit swimming during ingestion. However, because some swimming episodes can be elicited even during distention, it is unlikely that distention is inhibiting the P cells in the same manner as ingestion. Rather, distention is thought to target the maintenance of swimming rather than its initiation. Surgical removal of either the leech body wall plus gut tissue or gut tissue alone, suggests that the stretch receptors sensitive to feeding-induced distention are likely to be located in the body wall. Probable candidates for these receptors are the previously described stretch receptors (Blackshaw and Thompson, 1988; Cang et al., 2001; Friesen and Kristan, 2007) that help entrain the leech swim central pattern generator (Blackshaw and Thompson, 1988; Cang et al., 2001; Friesen and Kristan, 2007).

Although the above-described study (Misell et al., 1998) found little to no swimming within an hour following a full blood meal, a more recent report demonstrated that swimming can be induced post-feeding in some conditions (Claflin et al., 2009). Why the difference? One possibility is procedural differences: Misell et al. stimulated electrically with a train of pulses with fixed duration and amplitude, whereas Clafin et al. used mechanical stimulation. Additionally, the weight gain reported by Claflin et al. is substantially smaller (∼500%) than the ∼900% reported in other studies (Lent, 1985; Gaudry et al., 2010); the smaller distention might allow some maintained expression of swimming. Finally, Misell et al. compared swimming and crawling probabilities, whereas Claflin et al. focused solely on swimming. Regardless of this discrepancy, Claflin et al. found that distention through feeding profoundly affected the mechanics of leech swimming (Figure 3D). Immediately following ingestion, the speed of swimming was reduced by 25% and did not return to pre-feeding levels until the 10th day post-feeding. This decrease in swim speed was accompanied by decreases in the cycle frequency and the stride length (defined as the distance traveled in one swim cycle) of a swim cycle. Together all the data obtained from recently fed leeches suggests that swimming performance is negatively altered for an extended duration, biasing the leech’s behavior toward crawling rather than swimming.

Feeding affects not only locomotion in the leech but also the animal’s temperature preferences (Petersen et al., 2011). Prior to feeding, leeches acclimated to 21°C will settle into cooler waters below 15°C when placed in a temperature gradient (Figure 3E). Feeding shifts the leeches’ preference toward warming temperatures up to 24°C 1 day after feeding and elevated temperature preferences persist for up to 10 days. This phenomenon, termed post-prandial thermophily, is thought to aid animals in the digestion of their meal and has been extensively studied in reptiles (Sievert et al., 2005; Tsai and Tu, 2005; Bontrager et al., 2006; Stuginski et al., 2011). The study by Peterson et al. is likely the first to report such behavior in an invertebrate and it would be highly interesting to see if other obligate sanguivores such as the tick (*Ixodidae*) described above show similar behavior.

## Conclusion

**Figure 4:**
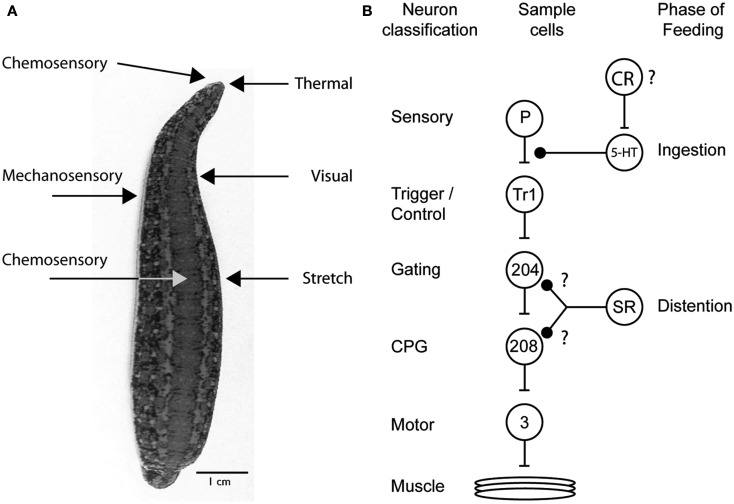
**Sensory receptors and targets involved in the leech’s decision to feed**. **(A)** Sensory receptors implicated in feeding based on behavioral experimentation. Chemosensory and thermal receptors on the dorsal lip are used to determine whether to attempt to feed on a potential food source. Additional chemosensory receptors sample the food in the gut and determine whether feeding will continue or cease. Visual and mechanosensory receptors located in the body wall allow the leech to orient into water waves to find their point of origin and thus likely prey. Stretch receptors in the gut of the leech serve to terminate feeding once a full meal has been ingested. **(B)** Diagram summarizing the multiple ways that leech feeding is known to inhibit the swimming circuit. The circles represent cell populations; the letters and numbers inside the circles indicate one identified neuron from that population type. The lines ending in bars represent excitatory connections, and those ending in solid black circles represent inhibitory connections. The diagram shows the excitatory, feedforward nature of the circuit but does not show the inhibitory interactions among the CPG neurons and between particular motor neurons. The inhibition from ingestion arises from an unknown source, probably chemical sensory pathways; it inhibits the P cell terminals via an unidentified serotonergic neuron. The actions of distention likely originate from stretch receptors in the body wall and target either the gating neurons or CPG neurons. The inhibition of cell 204 is speculative but consistent with an increase in swim period and a cessation of swimming behavior. Because leech stretch receptors hyperpolarize during stretch, the excitation of cell 208 may reflect the removal of inhibition rather than direct excitation. The swim circuit connections have been identified previously (Kristan et al., 2005). P, pressure mechanosensory P cell; Tr1, trigger neuron 1; 204, gating neuron 204; 208, CPG neuron 208; 3, dorsal longitudinal muscle excitatory motor neuron 3; SR, stretch receptors; CR, chemosensory receptor; speculated to encode distention; ?, potential connections.

Decades of research on the feeding behavior of the medicinal leech have revealed the complex interactions between neuromodulators, sensory receptors, and the downstream targets that influence how the medicinal leech controls feeding behavior (Figure 4A). Why are so many different mechanisms used just to perform one behavioral act? The research described in this review clearly illustrates just how complex decision-making processes are and how even the most mundane task requires several “check points” to ensure that the proper behavior is being performed and that competing behaviors are blocked out (Figure 4B). First, the right cues need to be detected. The leech relies on its keen thermal and chemoreception for this. Appetitive stimuli elicit feeding behaviors and aversive stimuli do not. However, once feeding has initiated, the leech now relies on a second check point to ensure that it has not made a mistake. These are the internal chemoreceptors located in its gut. This theme of multiple check points and circuits that can be recruited independently occurs throughout the decision to feed. During ingestion chemoreceptors drive serotonergic neurons that ultimately inhibit P cells and mechanosensory input into the leech ganglion. This prevents the initiation of behaviors like swimming. As the leech ingests blood, distention activates stretch receptors in the body wall decrease activity in the circuitry that maintains swimming, presumably in the system that activates the central pattern generator (Gaudry and Kristan, 2010). This design allows the nervous system to shut down all competing mechanosensory behaviors while the leech is feeding and allows most behaviors to come back online post-ingestion. However, because distention-mediated suppression of swimming can be recruited independently of ingestion, swimming remains inhibited long after the feeding bout has terminated.

How is decision-making in the leech similar to what is observed in mammalian nervous systems? Studying how a leech chooses to feed rather than respond to mechanosensory stimuli has revealed three majors principles that are also found in mammalian systems; sensory gating of information, distributed targets of decision circuits, and decision modules that can be recruited independently across tasks.

### Sensory gating

The mechanism used by a feeding leech to turn off all mechanosensory-induced behaviors (by serotonin-mediated pre-synaptic inhibition of the mechanosensory afferent terminals) is also found during modulation of pain in the mammalian nervous system. All pain afferents that enter the spinal cord are pre-synaptically inhibited by 5-HT and norepinephrine (Yoshimura and Furue, 2006) and this analgesic effect is prolonged by the action of endogenous opioids in the same pre-synaptic terminals, under a variety of behavioral conditions (Fields, 2007). For instance, in mice, just the presence of a cat evokes an analgesic effect (Kavaliers and Colwell, 1991). In general, when a mammal is performing a biologically important behavior (e.g., hiding, fighting, copulating, feeding), it often completes that behavior while ignoring stimuli that are painful or even injurious (Fields, 2007). This “gating out” of painful inputs is a mechanism for deciding “do not respond” to a sensory stimulation. The greater complexity in the mammal (i.e., three transmitters to “gate out” the pain, rather than a single one in the leech) may reflect a wider diversity of behaviors that modulate sensory inputs in the mammal, or it may mean that there will be additional modulatory substances and pathways to be found in the leech nervous system. In addition, this gating mechanism is not unique to mechanosensory inputs: similar examples of sensory gating have been found in the auditory (Krause et al., 2003) and olfactory (Murakami et al., 2005) systems of mammals.

### Distributed targets

In both leeches and vertebrates, decision-making is distributed across various regions of their brain. In mammalian pain modulation, for instance, the μ-opioid receptor responsible for pain suppression is expressed at every known supraspinal component of the pain modulating pathway, including the insular cortex, amygdala, hypothalamus, periaqueductal gray, dorsolateral pontine tegmentum, rostral ventromedial medulla, and the spinal cord dorsal horn (Fields, 2004). Thus pain is likely to be inhibited at several loci, analogous to how swimming is inhibited by satiety signals at multiple points in the leech.

### Decision modules

Like those in the leech, vertebrate decision-making circuits are modularized, with particular tasks performed by different brain regions that can be recruited independently. For instance, when a monkey compares two successive vibrating tactile stimuli, its brain encodes the sensation, stores the information, compares the two stimuli, and reports the decision. This complex series of actions are performed by different circuits for each component (Romo and Salinas, 2003): primary somatosensory cortex (S1) encodes the sensory stimuli; the prefrontal cortex (PFC) and secondary somatosensory cortex (S2) hold the signal in working-memory; at least part of the comparison between the two stimuli occurs in S2; and, finally, motor movements are initiated in the primary motor cortex (M1). The components of this highly distributed decision process can be recruited for other tasks; for example, the PFC is also used in making visual discriminations (Miller et al., 1996; Romo et al., 1999).

The similarities between decision-making circuits in leeches and mammals demonstrates the general usefulness of these broad concepts and illustrates how highly evolved invertebrate and vertebrate brains can use similar mechanisms to perform similar tasks.

## Conflict of Interest Statement

The authors declare that the research was conducted in the absence of any commercial or financial relationships that could be construed as a potential conflict of interest.
